# Editorial: New Therapies in the Field of Rheumatology

**DOI:** 10.3389/fphar.2019.01604

**Published:** 2020-01-28

**Authors:** Cecilia Beatrice Chighizola, Per-Johan Jakobsson, Maria Gerosa

**Affiliations:** ^1^ Experimental Laboratory of Immunorheumatological Researches, Unit of Allergology, Immunology and Rheumatology, IRCCS Istituto Auxologico Italiano, Milan, Italy; ^2^ Rheumatology Unit, Department of Medicine, Karolinska Institute, Solna, Sweden; ^3^ Department of Clinical Sciences and Community Health, University of Milan, Division of Clinical Rheumatology, ASST Istituto Gaetano Pini - CTO, Milan, Italy

**Keywords:** treatment, rheumatic diseases, biologic DMARDs, conventional DMARDs, pathogenesis

Rheumatology is a relatively recent medical specialty: the term “rheumatology” was coined by two American physicians only in 1940 (Rodnan, 1977). Approximately five decades after, rheumatology experienced a great advancement: biological agents became commercially available, revolutionizing the therapeutic approach to patients with systemic autoimmune diseases. The first biological compounds introduced in the market neutralize tumor necrosis factor alpha (TNFα), a key molecule in the pathogenesis of inflammatory arthritides. Despite the incontrovertible evidence on the efficacy of TNF-inhibitors, there are still many aspects that warrant clarification in order to optimize the therapeutic management of patients. Some of these critical issues are discussed in the Research topic entitled “New Therapies in the Field of Rheumatology”. Laganà et al. investigate whether gender affects the response to TNF-inhibitors among subjects with spondyloarthropathies or inflammatory bowel diseases (IBD), evincing a higher discontinuation rate of adalimumab but not infliximab in women with IBD. Even body weight might impact anti-TNFα efficacy, as demonstrated by Giani et al. In a cohort of 110 children with juvenile idiopathic arthritis, the remission rate is lower among obese patients, both for conventional disease modifying anti-rheumatic drugs and TNF-inhibitors. The accumulating experience with biological agents has also led to a better elucidation of adverse events. In particular, paradoxical immune-mediated inflammatory reactions, which consist in the onset or the exacerbation, during anti-TNF treatment, of manifestations commonly responding to biologics, are increasingly recognized. As discussed by Garcovich et al., they frequently involve the skin, mainly with a psoriatic presentation. Paradoxical reactions should be adequately accounted by clinicians as, although rare, are regarded as an important cause of biological discontinuation. With the third millennium, the rheumatology community has welcomed several new biologicals, such as anakinra and belimumab. Anakinra is a recombinant form of human protein interleukin (IL)-1 receptor antagonist (IL-1Ra), and has been used in adult onset Still’s disease (AOSD). Unfortunately, IL-1 blockade is not effective in all subjects, and in some cases loss of efficacy supervenes. It would be crucial to define the long-term efficacy of anakinra in AOSD, potentially identifying predictors of response. In a multi-centre study on 141 AOSD subjects, Vitale et al. (2019) report a retention rate at −60 and −120 months of 55.2 and 39.5%, respectively. In this study, only the number of swollen joints at baseline is predictive of secondary inefficacy of anakinra, an observation that might guide clinicians in the choice of treatment and follow-up of patients. Belimumab is the only biological agent approved for systemic lupus erythematosus (SLE). By targeting soluble B lymphocyte stimulator (BLyS, also known as BAFF), this monoclonal antibody modulates B cells, reducing the survival and the differentiation of B lymphocytes (Samotij and Reich, 2019). Regola et al. propose the usefulness of B cells immunophenotyping during belimumab therapy to monitor the response to treatment. In this study on 14 lupus patients receiving belimumab, a significant association between the decrease of B cells total number at 6 months and SLEDAI-2K improvement at 12 months emerges. More recently, the therapeutic armamentarium reserved to patients with psoriatic arthritis has been expanded significantly, thanks to the interests on the IL-23/IL-17 axis. IL-17 is a major pathogenic player in PsA, and IL-23 is upstream of IL-17. As reviewed by Sakkas et al., several pharmaceutical agents targeting the IL-23/IL-17 axis have been developed, with two compounds being already approved for PsA by regulatory agencies (secukinumab and ustekizumab).

The great progresses matured in the field of rheumatology thanks to the introduction of biological therapies do not imply neglecting the potential beneficial effects of conventional drugs. Achievement in clinical management of patients might be obtained by optimizing current treatments thanks to real-world evidence or by expanding the indications of drugs used in non rheumatological settings. This is the case of viscosupplementation in symptomatic hip osteoartrhritis. De Lucia et al. retrospectively assess the effectiveness and the tolerability of U.S.-guided intra-articular injection of hyaluronic acid. In a cohort of 122 patients, viscosupplementation reduces pain, intake of pain killers and joint stiffness, improving hip functionality. Benefits improve over 12 and 24 months, suggesting an additive effect of repeated injections. Sirolimus is a drug routinely used for the prevention of transplant rejection; by targeting the mammalian target of rapamycin (mTOR), sirolimus inhibits antigen-induced T cell proliferation and increases the number of circulating regulatory T cells. mTOR exerts a pivotal role in pathogenesis of lupus, a condition warranting novel therapeutic tools due to the high morbidity and mortality burden. Unsurprisingly, sirolimus has been tested as treatment for SLE. In a retrospective observational study on 27 patients with mildly active non-renal SLE, a Swedish group reports the efficacy of sirolimus on musculoskeletal manifestations, in particular arthritis and tendinitis (Eriksson et al.). Similarly, aminaphtone is a synthetic derivative of 4-aminobenzoic acid which has been widely used in case of microvascular impairment. Researchers from University of Genoa, Italy, evaluate the response to aminaphtone in a cohort of 46 patients with active Raynaud’s phenomenon. This six-month study demonstrates that aminaphtone increases skin blood perfusion and improves clinical symptoms due to Raynaud’s phenomenon, a condition still difficult to treat (Ruaro et al.). It can be also envisaged that, in the near future, a better diagnostic assessment of Raynaud’s phenomenon might guide treatment choices (Ruaro et al.).

Rheumatological diseases yet acknowledge a poorly clarified aetiopathogenesis: a further elucidation of pathogenic mechanisms will hopefully guide the development of future therapeutic tools. The present Research Topic includes two studies investigating the pathogenic role and the potential pharmacological relevance in rheumatoid arthritis (RA) of matrix metalloproteinases (MMP), endopeptidases deputed to degradation of extra-cellular matrix and cleavage of non-matric peptides. Horvárth et al. show in a mouse model of chronic arthritis the increase activity of all MMP isoforms in the joints. They also explore the effects of a broad-spectrum inhibitor of MMP, doxycycline, administered at a subantimicrobial dose. Doxycycline does not influence clinical signs, mechanical hyperalgesia and joint function. However, a worsening of bone microarchitectural lesions emerges, but such effect is most probably independent of MMP inhibition. Conversely, a Chinese research group reports that astragalin, a flavonoid extracted from leaves of persimmon and green tea seeds, effectively prevents the evolution of synovial inflammation and joint destruction in a murine model of collagen-induced arthritis (CIA), and such protective effect is partly mediated by the inhibition of MMP. Astragalin exerts an even broader action: it down-regulates pro-inflammatory cytokines in chondrocytes and synovial cells from CIA mice and inhibits p38 and JNK in MH7A cells treated with TNFα (Jia et al.). Furthermore, this issue provides insights into the pathogenesis of SLE: Furini et al. ascertain the role of P2X7R in a cohort of 48 lupus patients. P2X7R is an extracellular receptor for adenosine triphosphate, which is expressed by all immune cells. Upon activation, P2X7R guides the assembly of NLRP3 inflammasome, which in turn leads to the release of IL-1β and IL-18 and, indirectly, of additional cytokines involved in SLE pathogenesis. P2X7R expression is reduced in PBMCs from SLE subjects, even though there is no significant correlation between P2X7R activity and disease activity or duration, and experiments exclude a P2X7R role in driving the inflammatory response. These observations exert obvious pharmacology implications, as several drugs target IL-1β. Lastly, Yacoub and Schulke propose a novel model to explain the regulation of T cells by apoptotic cells, previously thought to be “immunologically null”. According to their theory, in a non-inflammatory context, apoptotic cells induce a immunosuppressive or tolerogenic response via CD80-CTLA4 coinhibitory signal to T cells, thus overriding costimulatory signals and ultimately counteracting T cell activation. Thanks to an extensive literature revision, these authors identify systemic autoimmune conditions in which this immunomodulatory model might be of therapeutic importance.

As a whole, the present Research Topic clearly pictures the complexity of the pharmacological approach in rheumatology. Despite the many progresses in the management of rheumatological diseases, clinicians are still far to offer optimal options to patients: the rates of treatment refractoriness are still high, side effects of drugs might be serious and costs are often burdensome. It is undeniable that much still remains to do, but the tremendous efforts of the research community give hope that many new advancements will be soon accomplished.

## Author Contributions

CBC, P-JJ and MG wrote the manuscript.

## Conflict of Interest

The authors declare that the research was conducted in the absence of any commercial or financial relationships that could be construed as a potential conflict of interest.
